# Labial Adhesion Surgery Prior to Vaginal Delivery in a Patient With Vulvar Lichen Planus Causing Labial Adhesion: A Case Report

**DOI:** 10.7759/cureus.74949

**Published:** 2024-12-02

**Authors:** Tomonori Taniguchi, Megumi Sasaki, Kojiro Segawa, Takeo Otsuki

**Affiliations:** 1 Department of Obstetrics and Gynecology, Sendai City Hospital, Sendai, JPN; 2 Department of Dermatology, Sendai City Hospital, Sendai, JPN

**Keywords:** inflammatory disease, labial adhesion, pregnancy, vaginal delivery, vulvar lichen planus

## Abstract

Lichen planus is an inflammatory disease that affects the skin and mucous membranes, and although rare, it can manifest in the vulvar region. Vulvar lichen planus can occur in women of reproductive age and may cause vulvar adhesion, potentially complicating examinations and delivery during pregnancy. We report a case in which a pregnant woman with vulvar adhesion due to vulvar lichen planus successfully delivered vaginally following labiaplasty.

The patient, a 31-year-old primigravida, was initially diagnosed with vulvar lichen planus approximately three years before her first visit to our hospital. Two months prior to her visit, she conceived through artificial insemination after letrozole administration. At her initial visit, her labial adhesion left only a small opening at the vaginal introitus, barely allowing the insertion of the little finger. At 12 weeks and five days of gestation, a labiaplasty was performed under spinal anesthesia. Postoperative treatment included topical steroids and tacrolimus ointment, leading to a full-term vaginal delivery. The estimated blood loss during delivery was 470 mL.

In cases where vaginal delivery may be challenging due to conditions such as labial adhesion, performing labiaplasty during pregnancy may facilitate a normal birth.

## Introduction

Lichen planus is an inflammatory disease that affects the skin and mucous membranes. Although lesions commonly occur in the oral cavity, vulvar lichen planus, while rare, has been estimated to affect approximately one in 4,000 women. Vulvar lichen planus is an immunologically mediated disease of the genitalia, and it has a broad spectrum of clinical presentation, ranging from diffuse erythema to severe erosions or hyperkeratotic plaques, and it may be accompanied by scarring and loss of the normal vulval architecture. While the application of topical steroids remains the mainstay of treatment, topical calcineurin inhibitors can be considered. Additionally, vulvar lichen planus has been noted to have the potential to progress to malignant disease, making regular follow-up necessary [1]. Vulvar lichen planus can affect women of reproductive age [2], potentially complicating pregnancy examinations and delivery due to labial adhesions. However, reports on pregnancies complicated by vulvar lichen planus are scarce. We report a case where a pregnant woman diagnosed with vulvar lichen planus during pregnancy underwent labiaplasty and was able to deliver vaginally. Informed consent was obtained from the patient for publication of this case report and accompanying images. 

## Case presentation

**Figure 1:**
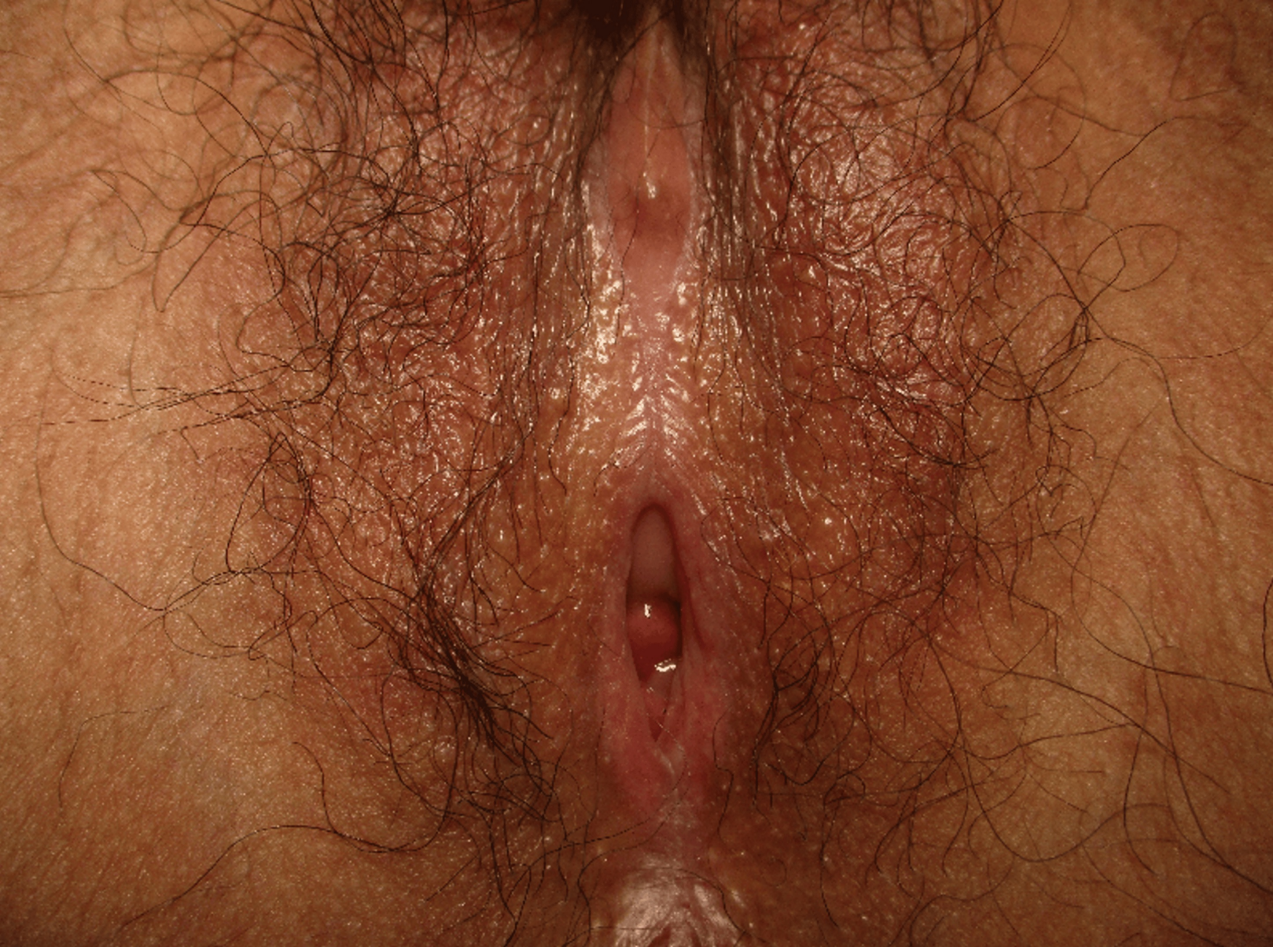
Findings upon vulvar examination at the time of initial consultation. A fusion of the labia was observed extending from the clitoris to the labia minora and labia majora, with the vaginal opening being reduced to barely allow the insertion of the little finger.

The patient, a 31-year-old primigravida, initially visited a local plastic surgery clinic approximately three years before her first visit to our hospital, presenting with pruritus and pain in the vulvar region. Redness and edema were observed in the transition area of the vaginal mucosa, and a skin biopsy confirmed the diagnosis of vulvar lichen planus. Her symptoms gradually improved, and she was advised to conduct vaginal massage during follow-up. However, the labial adhesion progressed, making sexual intercourse impossible, and she eventually discontinued follow-up visits on her own, although genital pain and discomfort persisted. Two months before her initial visit to our hospital, she visited a local clinic with a desire to conceive, and pregnancy was achieved through artificial insemination following letrozole treatment due to male infertility. Vulvar examination at the time of her initial visit to our department at 10 weeks and four days of gestation showed that her labial adhesion left only a small opening in the vaginal introitus, which could barely accommodate the insertion of the little finger (Figure 1).

**Figure 2:**
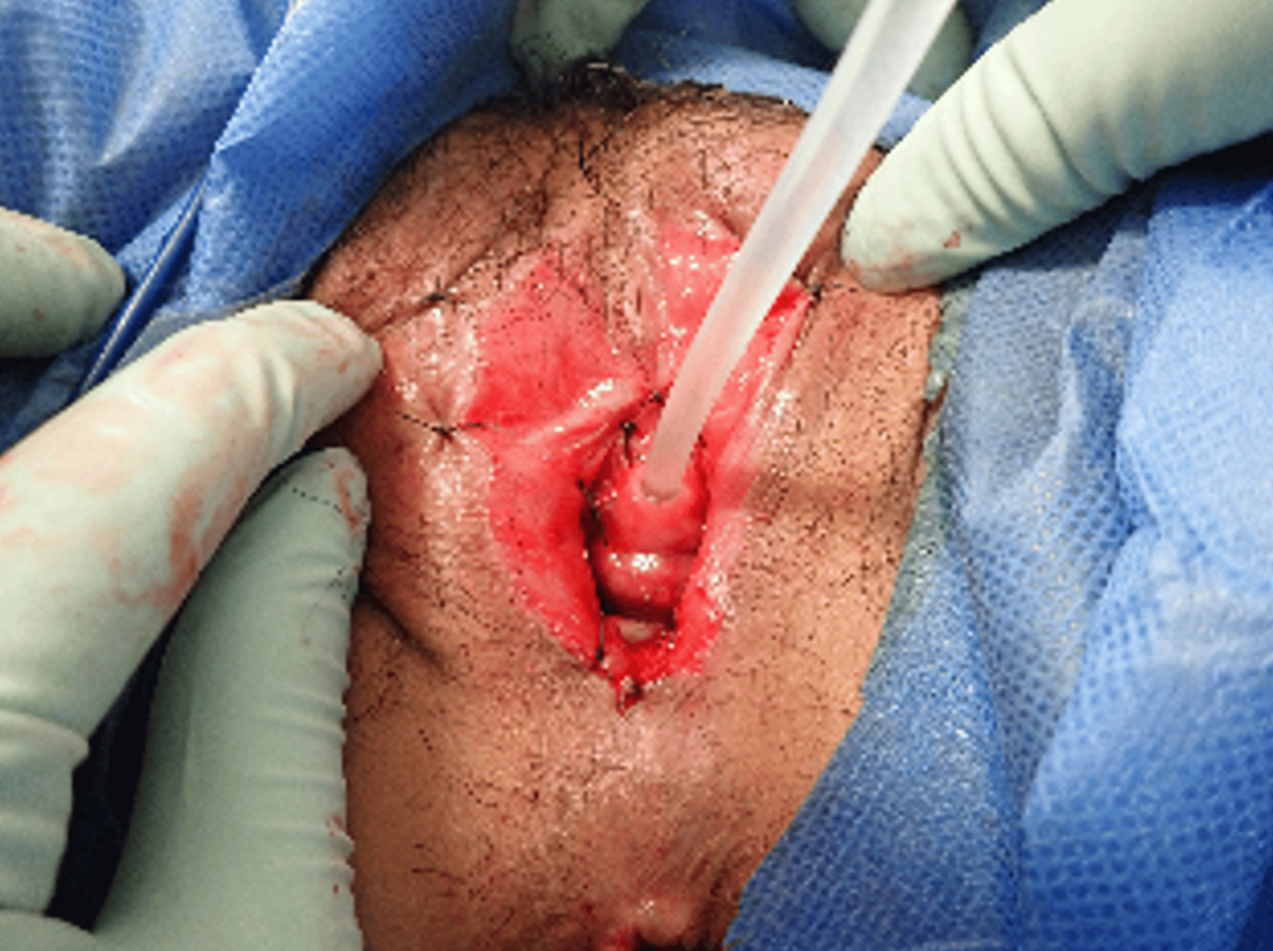
Findings upon vulvar examination immediately after labial adhesion surgery. The fused labial area had been bluntly dissected, and the left and right labia majora had been fixed to prevent re-adhesion.

We weren’t able to do transvaginal ultrasonography, so transrectal ultrasonography was performed to confirm fetal heartbeat, a crown-rump length of 45 mm, and a gestational age consistent with the fetal measurements. We planned to perform labiaplasty under spinal anesthesia. The patient was also referred to our dermatology department for concurrent follow-up. At 12 weeks gestation, labiaplasty was performed under spinal anesthesia using blunt dissection with manual techniques. To prevent re-adhesion, the labial edges were sutured with a 3-0 vicryl®︎ suture, ensuring adequate vaginal opening (Figure 2).

**Figure 3:**
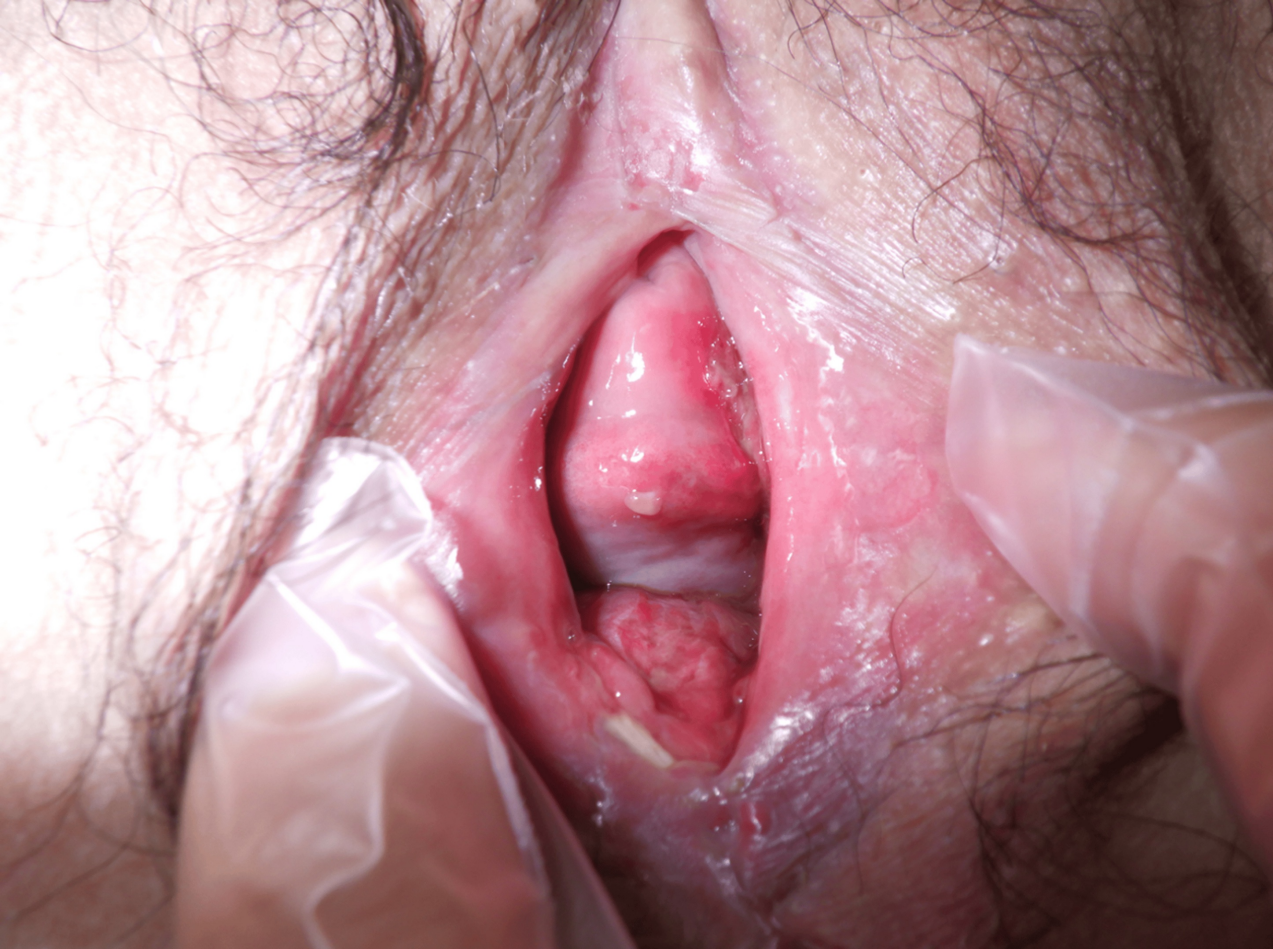
Findings upon examination of the perineal area at the time of discharge from the hospital. After vaginal delivery of the fetus, the vaginal opening was observed to be sufficiently open. A whitish lesion was observed on a part of the vaginal wall, but vaginal healing was uneventful.

The entire surgical procedure lasted 13 minutes with no blood loss. Postoperatively, the patient was instructed to manually separate the labia during urination, defecation, and bathing, and was prescribed 2% xylocaine jelly. She was discharged the next day. One week postoperatively, slight re-adhesion near the external urethral meatus and clitoral area required manual separation. Transvaginal ultrasonography then became possible without any difficulty. At 14 weeks of gestation, the surgical site appeared white, indicating a possible recurrence of lichen planus. It was revealed that she had not applied the prescribed 0.05% clobetasol propionate ointment, and so she was advised to resume its use. In the following week, re-adhesion of the labia minora, clitoris, and labia majora was noted from the anterior commissure to the clitoris. At her 20-week prenatal checkup, the fetal evaluation results were unremarkable, and transvaginal ultrasonography was feasible, although a medium-sized Cusco speculum was barely insertable. At 22 and 24 weeks, using a medium-sized Cusco speculum during examination caused a tear in the hymenal ring and vaginal wall at the 3 and 9 o’clock positions, resulting in minor bleeding that ceased spontaneously. At 27 weeks and five days, the vaginal wall appeared white, indicating possible exacerbation of the lesion, and administration of 0.03% tacrolimus hydrate ointment was initiated. Subsequent follow-up revealed no further lesion exacerbation, and no bleeding was observed during speculum examination. Given her stabilized condition, which allowed for sufficient healing of the inflammation and improvement in vaginal extensibility, we discussed the mode of delivery with the patient and her dermatologists, considering the risk of vaginal lacerations, and decided to start planning for a vaginal delivery trial. At 39 weeks of gestation, she was admitted to our hospital due to spontaneous labor onset, and on day one of being admitted, she had a vaginal delivery with a 5 o’clock episiotomy. The newborn weighed 3,005 g with Apgar scores of 8, 9, and 9 at one, three, and five minutes, respectively, with an umbilical artery pH of 7.258, and no abnormalities were noted. Cervical lacerations were absent, and only minor abrasions were noted on the vaginal wall, which did not require suturing. The perineal laceration at 5 o’clock was second-degree and was sutured with continuous 3-0 vicryl®︎ suture stitches as usual. Blood loss during delivery was 470 mL. Postpartum, tacrolimus ointment was temporarily discontinued. On postpartum day five, at the time of discharge from the hospital, no complications were observed, and she was discharged (Figure 3).

One month after delivery, wound healing was satisfactory, although signs of lichen planus persisted within the vagina, and the patient continues to receive outpatient follow-up at our department approximately once every three months for the risk of vulvar cancer.

## Discussion

Lichen planus is a relatively common disease affecting the skin and mucous membranes throughout the body. Oral lichen planus is the most common form, with a prevalence of about 1-2% among those aged 15 and older. The condition involving both oral and genital lesions has been classified as vulvovaginal-gingival syndrome and is rare [3]. Lichen planus tends to affect women more often than men, especially 50- to 60-year-old females; however, it can also manifest in the vulvar region of women of reproductive age, potentially co-occurring with pregnancy. Vulvar lichen planus during pregnancy can lead to complications such as vaginal irritation, antepartum bleeding, and postpartum hemorrhage [2]. Therefore, it is essential to control the lesions if vulvar lichen planus is present during pregnancy. Topical steroid ointments are commonly used as an initial treatment, offering the advantage of easy local application. A high-potency steroid ointment is generally applied for two to three months, followed by maintenance therapy to prevent recurrence. Systemic steroid therapy or tacrolimus may be considered as a second line of treatment [4,5].

In this case, administration of a topical steroid ointment alone was insufficient to prevent the recurrence of labial adhesion. Hence, treatment with a topical tacrolimus ointment was added, leading to good clinical outcomes. There have been few reports of vulvar lichen planus during pregnancy, and no reports, until now, of vaginal delivery following labiaplasty during pregnancy. Franz et al. reported a case of a pregnant woman with two prior vaginal deliveries who managed her condition with intravaginal hydrocortisone. Despite achieving a vaginal delivery, bleeding from the lichen planus lesions in the vagina persisted after birth, necessitating the application of hemostatic sutures in the operating room [2]. Bleeding during and after delivery can be a significant concern for vaginal deliveries in patients with lichen planus involving the vulva and vagina. It is essential to confirm that lichen planus lesions are well-controlled and not prone to excessive bleeding to determine whether vaginal delivery is feasible for pregnant women with vulvar lichen planus. In the case described here, severe labial adhesion initially made vaginal delivery unlikely; hence, labiaplasty was carried out. Treatment with topical steroids and tacrolimus hydrate ointments was used to manage her condition after surgery.

Tacrolimus has the potential to traverse the placental fetal-maternal barrier, potentially causing fetal adverse effects. The use of tacrolimus during pregnancy carries risks of low birth weight, preterm birth, transient neonatal hyperkalemia, and neonatal renal impairment [2]. In the present case, tacrolimus was used topically rather than systemically to minimize fetal exposure. Furthermore, our country lacks ointments containing immunosuppressants other than tacrolimus or steroid preparations. This makes tacrolimus advantageous as it is available in an easy-to-use ointment form. Due to the potential for severe inflammation and adhesion in the vulvar region and concerns about laceration healing after vaginal delivery, a cesarean section was also considered. However, following labiaplasty, the condition of the patient was sufficiently controlled, allowing for vaginal delivery. Cases like this, where labial adhesion due to lichen planus is severe, suggest that labiaplasty and subsequent treatment may enable vaginal delivery, making labiaplasty a viable treatment option for women of reproductive age even during pregnancy.

Labial adhesion is thought to develop in patients with underlying inflammatory vulvar lesions, particularly in cases of abstinence from intercourse or estrogen deficiency. Since pregnant women are typically assumed to be sexually active, labial adhesion is considered unlikely. However, cases of labial adhesion during pregnancy have been reported. In one such report, the patient did not engage in intercourse, achieving pregnancy via ejaculation near the labia by a sexual partner. This underscores the possibility that pregnant women may experience labial adhesion without intercourse. Additionally, in that case, the patient had a history of genital herpes, which contributed to labial adhesion. The patient experienced difficulty with urination due to labial adhesion and underwent labiaplasty during pregnancy, although the pregnancy was terminated at eight weeks as it was unintended [6].

Vulvar lichen planus carries a risk of malignant transformation. While vulvar squamous cell carcinoma occurs at a frequency of approximately 0.18 per 1,000 person-years, this rate increases significantly to 25.9 per 1,000 person-years in patients with vulvar lichen planus [1]. Due to this elevated risk, long-term follow-up is essential for patients with vulvar lichen planus [7], and prompt treatment is required if squamous cell carcinoma develops.

Gynecologists are generally accustomed to performing vaginal examinations, including for cases of labial adhesion. In the case described here, good clinical outcomes were achieved through initial treatment via plastic surgery, followed by concurrent management by dermatology, obstetrics, and gynecology departments. In treating genital lesions like labial adhesion, interdisciplinary collaboration among multiple departments, including gynecology, can potentially improve outcomes.

## Conclusions

This report presents the first case of a successful vaginal delivery following labiaplasty for vulvar lichen planus during pregnancy. In cases where vaginal delivery may be challenging, such as with labial adhesion, labiaplasty performed during pregnancy may facilitate a normal birth. In cases of labial adhesion, there is a possibility that the patient may first visit departments other than gynecology or dermatology, which are typically accustomed to examining the vulvar area. Therefore, when labial adhesion is caused by a dermatological disease, it is important for relevant departments, including gynecology, to collaborate in providing treatment.
